# Differences in the consolidation by spontaneous and evoked ripples in the presence of active dendrites

**DOI:** 10.1371/journal.pcbi.1012218

**Published:** 2024-06-25

**Authors:** Jannik Jauch, Moritz Becker, Christian Tetzlaff, Michael Jan Fauth

**Affiliations:** 1 Third Institute for Physics, Georg-August-University, Göttingen, Germany; 2 Group of Computational Synaptic Physiology, Department for Neuro- and Sensory Physiology, University Medical Center Göttingen, Göttingen, Germany; University College London, UNITED KINGDOM

## Abstract

Ripples are a typical form of neural activity in hippocampal neural networks associated with the replay of episodic memories during sleep as well as sleep-related plasticity and memory consolidation. The emergence of ripples has been observed both dependent as well as independent of input from other brain areas and often coincides with dendritic spikes. Yet, it is unclear how input-evoked and spontaneous ripples as well as dendritic excitability affect plasticity and consolidation.

Here, we use mathematical modeling to compare these cases. We find that consolidation as well as the emergence of spontaneous ripples depends on a reliable propagation of activity in feed-forward structures which constitute memory representations. This propagation is facilitated by excitable dendrites, which entail that a few strong synapses are sufficient to trigger neuronal firing. In this situation, stimulation-evoked ripples lead to the potentiation of weak synapses within the feed-forward structure and, thus, to a consolidation of a more general sequence memory. However, spontaneous ripples that occur without stimulation, only consolidate a sparse backbone of the existing strong feed-forward structure.

Based on this, we test a recently hypothesized scenario in which the excitability of dendrites is transiently enhanced after learning, and show that such a transient increase can strengthen, restructure and consolidate even weak hippocampal memories, which would be forgotten otherwise. Hence, a transient increase in dendritic excitability would indeed provide a mechanism for stabilizing memories.

## Author summary

Sequential activity of place cells in hippocampus has been found to be replayed during sleep. Such replay is believed to aid the consolidation of episodic memories. We here investigate replay of memories encoded by a feed-forward structure within a recurrent network as well as associated synaptic plasticity. Hereby, we vary both the external stimulation that triggers replay-sequences, as well as the excitability of active dendrites. With stimulation and highly excitable dendrites, we observe reliable replays and potentiation of both weak and strong synapses along the feed-forward structure. Without stimulation but with highly excitable dendrites, spontaneous replays emerge and only sufficiently strong synapses are further potentiated. Without highly excitable dendrites, the memory decays. However, with an intermediate period of increased dendritic excitability, the memory can be sufficiently restructured and strengthened to exhibit spontaneous replays and consolidation, even at lower dendritic excitability levels. These results indicate that a transient increase in dendritic excitability provides a mechanism both for restructuring and stabilizing hippocampal memories.

## Introduction

While animals explore their environment, place cells in the hippocampus give rise to sequential activity. Later on, during sleep and rest phases, this sequential activity is replayed, often in a time-compressed fashion [1, 2]. Such replays mostly occur during hippocampal activity complexes called sharp-wave ripples (SWRs) [3, 4]. SWRs and the associated replay of sequential activity patterns are believed to be essential for hippocampus-dependent memory consolidation [5–7]. Accordingly, the suppression of SWRs impairs [8] and their enhancement strengthens this kind of memory [9]. As an underlying mechanism, it is hypothesized that ripple activity provides a good basis for further synaptic plasticity, which then consolidates the memories. Hereby, both synaptic potentiation [10] as well as synaptic depression [11] have been associated with SWR activity.

The mechanism how SWRs are evoked and especially whether SWR-activity emerges spontaneously or whether it depends on input from other brain areas, is not completely clear. On the one hand, *in vivo* experiments indicate that input, for example from CA3, is driving SWRs in CA1 [12–14]. On the other hand, also slice preparations lacking this kind of input exhibit spontaneous SWRs [15–17]. Thus, both input-evoked and spontaneous ripples might coexist in the same network. Given the link between ripples and memory, we here want to clarify whether spontaneous and input-evoked SWRs have similar or different effects on plasticity and memory consolidation.

Moreover, also the active integration and depolarization of dendrites plays a critical role in SWR and the induction of synaptic plasticity [10, 18, 19]. For instance, dendrites exhibiting synaptic plasticity at their synapses have an increased excitability [20–23], which led to the hypothesis that dendrites hosting newly learned memories are more excitable and, thus, are primed for consolidation [24]. Following this hypothesis, we also examine the role of non-linear dendritic integration and its temporal changes for the neural activities and synaptic plasticity during SWRs—both spontaneous and input-evoked.

To better understand the interplay between all discussed processes, we rely on mathematical models.

A replay of sequential activity is commonly observed in models with temporally asymmetric Hebbian learning and can occur both as a consequence of cueing the first sequence element [25, 26], but also during spontaneous activity [27, 28]. In these models, correlated firing of neurons coding for one sequence element triggers the firing of neurons coding for the next sequence element. If neural activities are sparse, these populations can be non-overlapping, which effectively gives rise to a feed-forward structure through which activity propagates. This propagation, however, does not necessarily imply the presence of ripple-like oscillatory behavior. Another line of modeling studies has investigated oscillatory activities in recurrent networks. Ripple-like oscillations can, on the one hand, originate from an interconnected inhibitory population, which then gates the activity of excitatory cells (inhibition-first models, [29–32]). On the other hand, also recurrent connections between excitatory cells, in interaction with the excitatory-inhibitory feedback loop, may be the reason for ripple activity (excitation-first models, [32–35]). The latter mechanism typically relies on fast propagation of activity between the excitatory cells, which may happen due to electrical coupling through gap-junctions [32–36] or due to fast non-linear dendritic integration [36]. In the brain, most likely a mixture of all of these variants may be prevalent [37]. Note that in many computational models, ripple generation relies on the presence of external stimulation signals (but see [36, 38]), although some studies included other brain areas, which generate these inputs (other hippocampal areas, cortex, thalamus), and investigated the interrelation of activities between the brain areas [39, 40] as well as the learning related changes of these activities [41].

We base our work on a well-established computational model [42] that integrates active dendrites and explains learning, replay of memories and SWRs. In this model, episodic (sequence) memories are encoded by feed-forward structures which—upon replay—exhibit ripple-like activity triggering synaptic plasticity. Based on this model, in this study, we systematically vary the dendritic excitability as well as the putative input from other brain areas and evaluate the impact on the structure of the memory representations.

We find that the emergence of ripple-associated sequence replay and synaptic plasticity depends on reliable propagation of activity through the memory-related feed-forward structure, which, in turn, is enhanced by highly excitable dendrites. Under these conditions, networks with spontaneous and evoked ripples exhibit very different patterns of synaptic plasticity: In networks with spontaneous ripples and no input only a subset of the connections within the feed-forward structure stays strong and undergoes further LTP. Hence, a reduced backbone of the feedforward-structure remains. In networks that are also stimulated externally, initially weak synapses between neurons of the feed-forward structure are also potentiated. These synapses partly connect neurons with distant place fields, which were previously multiple steps apart in the feed-forward structure. Thereby, a generalized representation of the place field order in the feedforward structure emerges. We then demonstrate that a transient increase in dendritic excitability after learning—in combination with external stimulation—leads to a restructuring of the memory representation such that it gets reactivated even when dendrites become less excitable later-on. Thus both of the above mechanisms could be employed to consolidate and stabilize hippocampal sequence memories, as hypothesized in [24].

## Materials and methods

In order to built on previous results on forming sequence memories and the emergence of replay and ripple-activity, the model presented in the following as well as the value of the parameters largely follow Jahnke et al. [42] and are justified therein. The amendments that have been introduced in this work are explicitly laid out at the end of this section.

### Neuron model

We use conductance-based leaky integrate-and-fire neurons, whose membrane potential follows

$$\tau_{m}\frac{du_{i}\left(t\right)}{dt}=u_{\text{rest}}-u_{i}\left(t\right)+R_{m}I_{i}\left(t\right)+\zeta_{i}\left(t\right),$$

where *u*_rest_ = −65 mV is the resting potential, *τ*_*m*_ = 16 ms or 8 ms the membrane time constant for excitatory and inhibitory neurons respectively and *R*_*m*_ = 40 MΩ the membrane resistance. The term *ζ*_*i*_(*t*) is Gaussian white noise with 〈*ζ*_*i*_(*t*)〉 = 0 and $\langle \zeta_{i}\left(t\right)\zeta_{j}\left(\tilde{t}\right)\rangle =\sigma^{2}\delta_{ij}\delta \left(t-\tilde{t}\right)$, with *σ* = 3 mV. *I*_*i*_ is the current influx into neuron *i*, which is calculated as

$$I_{i}\left(t\right)=\sum_{j}g_{i,j}\left(t\right)\cdot \left(E_{j}-u_{i}\right)+I_{\text{den}}+I_{\text{BG}}.$$

Here, *I*_BG_ = 0.38 nA is the constant background current and *I*_den_ depicts currents from active dendritic processes (see below). The first term describes the current through synapses, which are characterized by their conductance *g*_*i*,*j*_ and reversal potential *E*_*j*_ of the respective synapse, which is either *E*_ex_ = 0 mV or *E*_inh_ = −70 mV depending on the type of the presynaptic neuron *j*. The conductances evolve according to

$$\tau_{g}\frac{g_{i,j}}{dt}=-g_{i,j}$$

with *τ*_*g*_ = 3 ms, and are increased by *w*_*i*,*j*_ after each presynaptic spike with a conduction delay *d*_syn_ = 3 ms, where *w*_*i*,*j*_(*t*) is the synaptic weight of the respective synapse.

If the membrane potential exceeds a threshold *θ* = −45 mV, it is reset to −65 mV and in-flowing currents are not considered throughout a 3ms refractory period.

### Active dendrites

We further include the possibility of dendritic spikes, if a sufficiently strong input occurs. For this, we check whether the sum of all excitatory conductances exceeds a threshold *g*_thres_ within a time window of 2 ms. The threshold is set at 7.27 nS for the networks with enhanced dendritic excitability and 10.17 nS in the networks without. If that threshold is exceeded, a dendritic spike current is emitted, which takes the shape

$$I_{\text{den}}=\Theta \left(t-t_{s;i}-d_{\text{den}})\sum_{k=1}^{3}A_{k}\;\text{exp}\;(-(t-t_{s;i}-d_{\text{den}}\right)/\tau_{k})$$

with amplitudes *A*_1_ = −55 nA, *A*_2_ = 64 nA and *A*_3_ = −9 nA as well as time-constants *τ*_1_ = 0.2 ms, *τ*_2_ = 0.3 ms and *τ*_3_ = 0.7 ms. Note that we calculate *I*_den_ only relative to the last dendritic spike at time *t*_*s*;*i*_.

### Network structure

We simulate a network comprising 480 neurons, which are separated into 80 inhibitory and 400 excitatory neurons. All neurons are sparsely connected through synapses with 8% probability between excitatory neurons (ex→ex), 10% between excitatory and inhibitory neurons (ex→in, in→ex) and 2% between inhibitory neurons (in→in). Weights are initially drawn from Gaussian distributions with means *μ*_*ex*→*ex*_ = 0.7 nS, *μ*_*ex*→*in*_ = 1.0 nS, *μ*_*in*→*ex*_ = 2.5 nS, and *μ*_*in*→*in*_ = 2.0 nS as well as standard deviations *σ*_*ex*→*ex*_ = 0.16 nS, *σ*_*ex*→*in*_ = 0.1 nS, *σ*_*in*→*ex*_ = 0.25 nS, and *σ*_*in*→*in*_ = 0.2 nS. Synapses between excitatory neurons undergo spike-timing-dependent plasticity and synaptic scaling (see below).

#### Sequence memory

**Fig 1:**
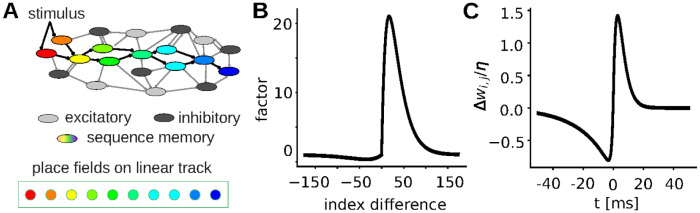
Schematics of the model used to investigate ripple activity and plasticity. (A) A predefined excitatory feed-forward structure was embedded in an excitatory-inhibitory network and its evolution with and without stimulation as well as with different sensitivities of nonlinear dendrites was tested. (B) Factor applied to the synaptic weights within the feed-forward structure depending on the index difference between post- and presynaptic neuron (spatial kernel). (C) Weight change depending on time difference between post- and presynaptic spikes (STDP-curve)

In order to exert better control over the feed-forward structure that has been demonstrated to emerge in the network during learning [42], we initialize networks with a predefined feed-forward structure. For this, we assume the excitatory cells with indices 100 to 330 correspond to place fields along a linear track (colored cells in Fig 1A). The connections emerging from neurons with indices from *M*_start_ = 100 to *M*_end_ = 299 are increased or decreased by a factor corresponding to a spatial kernel shown in Fig 1B. The kernel shape is estimated from [42]:

$$\begin{array}{c} \Delta w_{i,j}^{kernel}=\{\begin{array}{cc} \frac{1}{A_{\text{fw}}}\cdot [e^{-\frac{i-j}{21}}-e^{-\frac{i-j}{14}}]w_{i,j}, & \text{if}\;i\geq j\;\text{and}\;M_{\text{start}}\leq i,j<M_{\text{end}},\\ \frac{1}{A_{\text{bw}}}\cdot [-e^{-\frac{j-i}{42}}+e^{-\frac{j-i}{28}}]w_{i,j}, & \text{if}\;i<j\;\text{and}\;M_{\text{start}}\leq i,j<M_{\text{end}}, \end{array} \end{array}$$

where *A*_fw_ = 7.4 ⋅ 10^−3^ and *A*_bw_ = 0.2 are normalization constants for the strengthening of the feed-forward connections and the weakening of the backward connections, respectively.

### Synaptic plasticity and scaling

The synaptic weights of connections between excitatory neurons evolve according to a spike-time dependent plasticity rule that depends on the time distance between pre- and post-synaptic spikes Δ*t* in a smooth fashion (Fig 1C):

$$\begin{array}{c} \Delta w_{i,j}=\eta_{\text{STDP}}.\{\begin{array}{cc} {}[A_{+}\cdot k\left(\Delta t,\tau_{+,0}\right)-A_{-}\cdot k\left(\Delta t,\tau_{-,0}\right)]\;\text{exp}\;(\frac{-\Delta t}{\tau_{\text{STDP}}}), & \text{if}\;\Delta t\geq 0,\\ A_{+}\cdot \;\text{exp}\;(\frac{\Delta t}{\tau_{+,0}})-A_{-}\cdot \;\text{exp}\;(\frac{\Delta t}{\tau_{-,0}}), & \text{if}\;\Delta t<0, \end{array} \end{array}$$

with $k\left(\Delta t,\tau_{0}\right)=1+\Delta t\frac{\tau_{\text{STDP}}+\tau_{0}}{\tau_{\text{STDP}}\cdot \tau_{0}}$. Here, *η*_STDP_ = 0.12 is the learning rate, *τ*_STDP_ = 3 ms, *τ*_+,0_ = 1 ms and *τ*_−,0_ = 20 ms are the time-constants and *A*_+_ = 1.2 nS and *A*_−_ = 1.0 nS the amplitudes of the STDP-window.

Moreover, these synapses undergo synaptic scaling [43]. For this, we apply:

$$\Delta w_{i,j}=-\eta_{\text{SC}}\;v_{i}{\left(w_{i,j}/g_{*}\right)}^{2}$$

every 10 ms, where $\eta_{\text{SC}}=10^{-3}\text{nS}\;\cdot \;{\text{Hz}}^{-1}$ is the scaling rate, g_*_ = 1.0 nS is a normalization factor, and *v*_*i*_ is the current firing rate of the neuron estimated from the spike count in a moving window of length 500 ms. The non-linear dependence on *w*_*i*,*j*_ is necessary to counteract run-away potentiation due to the positive feedback between synaptic weights and post-synaptic activity that occurs for many Hebbian synaptic plasticity rules [43], like the STDP rule used in this study.

### Simulation paradigms

Initially, we compare four cases of networks: (i) networks without external stimulation and low dendritic excitability; (ii) networks without external stimulation and high dendritic excitability; (iii) networks with external stimulation and low dendritic excitability and (iv) networks with external simulations and high dendritic excitability.

When ripple activity is evoked by external stimulation, e.g. from another brain area, we stimulate a group of 50 neurons at the beginning of the feed-forward structure by scaling the noise with a sine-wave with an amplitude of 0.714, an offset of 1.071 and a varying frequency between 9 Hz and 14.5 Hz (changed every 500 ms).

For each of these cases, we simulate the above described network for 262 s. Hereby, we start by simulating the network without active dendrites for 1 s for equilibration. Then, we simulate the network for another 31 s without plasticity to gather statistics on the activity. Then a 200 s period with plasticity is simulated, after which plasticity is turned off again for 30 s to gather statistics on the activity. At this time-point plasticity has usually converged to a stationary value such that activity and connectivity after plasticity can be further investigated.

Secondly we simulate a plastic network which progresses between the described states. Here again we simulate a 1 s initialization period, followed by 199 s with active dendrites with enhanced excitability and external stimulation. Then, external stimulation is switched off, and the network is simulated for another 200 s. Finally, also the dendritic excitability is decreased and the network is simulated for another 200 s. Connectivity is tracked over time and especially each time before switching to another network state.

### Continuous Wavelet transform

To identify ripple like activity, we evaluate the spectral power in different frequency bands. For this, we used a continuous wavelet transform with the complex Morlet wavelet using the pywavelets package [44] and applied it to the summed activity of all cells. In specific, we analyzed frequencies from 70 to 300 Hz with wavelet scaling factors between 2.7 and 12, bandwidth *B* = 2.0 and center frequency constant *C* = 0.8 [45, 46].

### Reduced model

In a reduced model of the feed-forward structure, we investigate activity propagation using a single post-synaptic neuron receiving input from a varying number *N*_neur_ of pre-synaptic neurons. The spike trains of these neurons all have the same pairwise correlation coefficient *c* and firing rate *r*, which is varied throughout the experiments, and are generated using the multi-interaction method described in [47]. The parameters of the reduced model are the same as for the large-scale network simulations, if not stated otherwise. The reduced model is simulated for 30 s and the number of spikes, dendritic spikes and the change of synaptic weights are tracked.

### Comparison to Jahnke et al., 2015 [42]

In the following, we shortly list the main differences of the model used here and [42], which mainly arose due to the fact that we focus here on understanding the long-term consolidation of memories, which requires more computational resources for simulations.

- No simulation of learning: In this study we wanted to focus on the consolidation during sleep. Therefore, we wanted a to have more control over the weights in the memory structure after learning and decided to not explicitly simulate the learning process. Instead, we encoded the learned memory into the initial synaptic weights. For the weight changes, we drew inspiration from the strongest memory structure reported in [42], approximating the weights with a double-exponential kernel function (Fig 1B).
- No spatial structure: For simplicity, we chose to not simulate the spatial distribution of the neurons to obtain their conduction delays, but assume fixed, homogeneous delays for the signal transduction between each pair of neurons.
- Synaptic plasticity: We use a modified version of the STDP-rule from [42] and added synaptic scaling to prevent run-away potentiation due to repeated replays, which we encountered without.
- Network size: For fast and efficient exploration, we reduced the number of neurons in the network to 480.
- Inhibitory neurons: For simplicity and more efficient simulation, spiking threshold and conductance decay have been adapted to the excitatory ones. To make up for the slower dynamics, the fraction of inhibitory neurons has increased from 10% to 20% of the excitatory neurons.
- Background input: Instead of Poisson inputs to each neuron, we use a fixed background current and Gaussian white noise driving the membrane voltage to speed up simulation. Both were tuned to obtain asynchronous irregular activity.

## Results

We study the emergence of ripples and corresponding changes in connectivity in a recurrent network model with active dendrites, spike-time dependent plasticity and synaptic scaling. As our focus lies on the interplay between sleep-like activity and synaptic plasticity, we start our simulations after learning and initialize our sequential memory as a potentiated feed-forward structure in the network (Fig 1A and 1B), matching the one found in [42]. Hereby, two key components are varied: On the one hand, we investigate networks with a periodic external stimulation at the beginning of the feed-forward structure (sketched in Fig 1A), which supposedly evokes activity propagation (i.e., replays), as well as networks without this stimulation, in which replays can only emerge via the spontaneous activity of the network. This stimulation may correspond to inputs from other brain areas that arise through typical activity in sleep, but also to an optogenetic stimulation that has been used in some experiments [48, 49]. On the other hand, we varied the excitability of the dendrites, which ultimately regulates how likely a neuron spikes in response to an incoming spike and, thus, the likelihood that activity is propagated in the feed-forward structure. For simplicity, we initially chose to investigate one low-excitability and one high-excitability configuration in our simulations, which were chosen such that the high-excitability network exhibits spontaneous replays of the sequential memory represented by the feed-forward structure and the low one does not.

**Fig 2:**
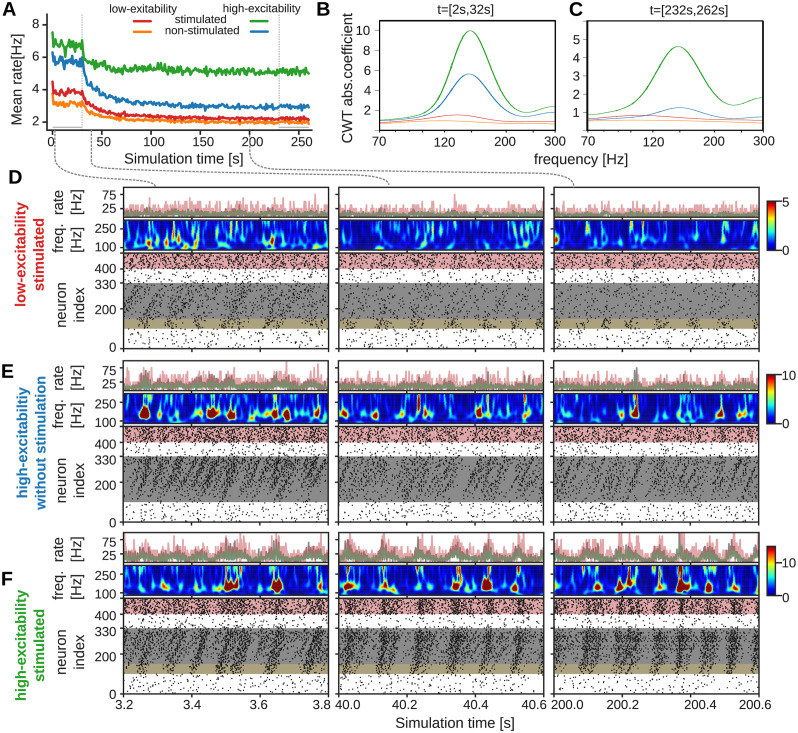
Ripple activity for spontaneous and stimulated replays. (A) Time-course of the mean firing rate for all neurons in different network configurations. (B-C) Absolute value of continuous wavelet transform coefficient over relevant frequency range averaged over 30 s before (B) and after rest phase (C). Ripple frequency is about 150 Hz. (D-F) Top panel: mean rates of feed-forward (gray) and inhibitory (red) neuron populations and all neurons (green). Middle panel: time-dependent continous wavelet transform. Bottom panel: Spike raster-plots of the different configurations at different time points. Left: before rest phase; Middle: rest phase; Right: after rest phase. Yellow boxes mark neurons with additional stimulation. (D) Low-excitability network with stimulation. (E) High excitability network without stimulation. (F) High excitability network with stimulation.

Thus, we investigate four network configurations: (i) low-excitability without stimulation (orange in Fig 2), (ii) low-excitability with stimulation (red) (iii) high-excitability without stimulation (blue) and (iv) a high-excitability with stimulation (green).

### Temporal evolution of network activity with spontaneous and input-evoked ripples

As a first step, we examined the neuronal activity before, during and after a sleep-like or rest period in each of these configurations. For this, we first simulated a 30 s initialization period during which plasticity was switched off. Secondly, we observed the evolution of activities when plasticity is switched on for 200 s, which we will refer to as the rest phase. Thirdly, another 30 s phase without plasticity is simulated to gather activity statistics.

We first assessed the mean firing rates emerging for all neurons in the different networks and find that both low-excitability configurations show similar low firing rates, whereas the stimulated ones retain a slightly elevated firing rate throughout and after the rest phase (Fig 2A). After plasticity is switched on, a drop in the activity in all networks occurs. This drop could be an indication that the initialized feed-forward structure representing a freshly learned memory is not completely stable. The unstimulated low-excitability network did not show replay activities and also has no power in the corresponding ripple-frequency bands around 150 Hz (orange, Fig 2C), such that we did not further investigate this model variant. However, examining the activities in the stimulated, low-excitability network on a finer scale (spike raster plots in Fig 2D), we observe a periodic replay activity in the network initialization phase, which is likely due to the periodic stimulation and spans over the whole feed-forward structure (grey area in Fig 2D, left, Fig A:A in S1 Appendix). At the beginning of the rest phase, we see that the replay activity becomes sparser (Fig 2D, middle), while after the rest phase only the stimulated neurons are periodically activated, but the activity does not propagate to the rest of the feed-forward structure (Fig 2D, right, and 2A). Accordingly, the quality of sequence replays declines strongly over time (Fig A:B in S1 Appendix).

Moreover, the decline in propagation also becomes visible in the frequency spectrum of overall network activity (Fig 2C): Whereas initially, the stimulated low-excitability network exhibits a peak in the ripple-associated frequencies around 150 Hz (left panel), this peak vanishes after the rest phase (right panel).

In contrast, the high-excitability networks show pronounced peaks for ripple frequencies both during initialization and after the rest phase. In terms of the mean firing rate, the unstimulated, high-excitability network exhibits a slightly lower rate during initialization but a much larger drop during the rest phase. When assessing the activity of the unstimulated network on a detailed level, we initially find frequent activity propagation along the feed-forward structure (Fig 2E, left, and Fig A:C in S1 Appendix). In contrast to the stimulated networks, this propagation starts spontaneously somewhere within the structure and propagates to the end (see Fig A:C, panel i in S1 Appendix). These (partial) replays become sparser during the rest phase (Fig 2E, middle and right), which also entails a decline in ripple frequency power (Fig 2C). However, as the activity still propagates to the end of the feed-forward structure in a temporally ordered fashion (Fig A:C in S1 Appendix), the quality of sequence replay remains high (Fig A:D in S1 Appendix).

In comparison, the stimulated high-excitability network initially exhibits both complete replays at the times of the periodic simulations as well as spontaneous replays that occur between the input-evoked replays (Fig 2F, left). During the rest phase, these spontaneous replays become sparser, while the evoked replays persist (Fig 2E, middle and right). In contrast to the stimulated low-excitability network, the propagation of the activity keeps reaching the end of the feed-forward structure, which explains the much higher mean firing rate. Hence, the combination of spontaneous and input-evoked ripples leads to stable ripple propagation along the feed-forward-structure inducing reliable replay. Yet, at the end of the rest phase, activity seems to propagate to the end of the feed-forward structure much faster, by skipping some neurons, such that the order of spiking does not correspond to the sequence anymore (Fig A:E in S1 Appendix), which entails a decrease in replay quality (Fig A:F in S1 Appendix).

Taken together, we thus find that a high dendritic excitability aids the propagation of activity within the feed-forward structure and is a key factor for the emergence of spontaneous sequence replays and, thus, ripple activity.

### Restructuring of memory representations is different for spontaneous and input-evoked ripples

In the next step we examine the evolution of the synaptic weights for different configurations.

**Fig 3:**
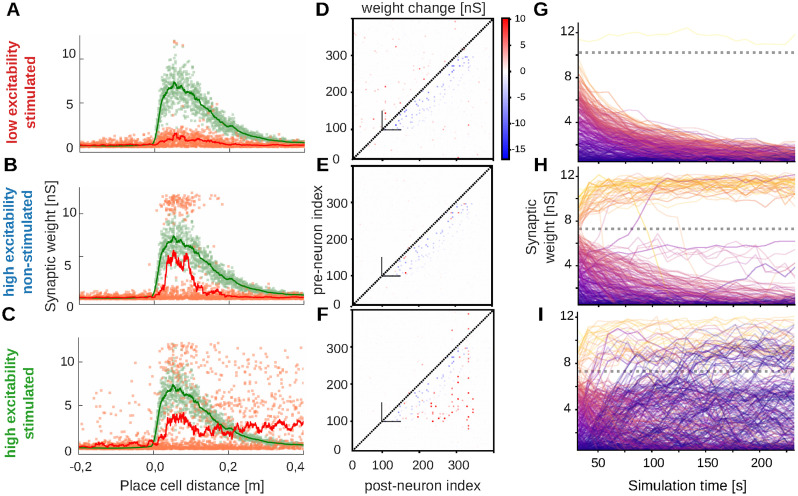
Synaptic weight changes induced by rest phase for different network configurations. (A-C) Synaptic weights before (green curve and points) and after rest phase (red curve and points) depending on the distance between place fields of post- and presynaptic neuron within the feed-forward structure. (D-F) Change of the synaptic weights during the rest phase depending on pre- and postsynaptic neuron index. Neurons 100–300 represent the feed-forward structure with strong initial synaptic weights above the main diagonal (dashed line). (G-I) Time courses for individual synaptic weights (initial weight indicated by trace color). Dashed lines mark the threshold for non-linear dendritic integration.

The initial feed-forward structure that represents a (sequential) memory was constructed by mapping each neuron to a place field along a linear track and strengthening the connections to neurons further down the track using a spatial-distance dependent kernel (Fig 1B, compare Fig 3A–3C, green curves).

In the same way, we can average the weights of connections with neurons that have the same spatial distance along the track and assess the resulting weight kernels after rest phase (red curves). For the stimulated, low-excitability network, we find that the weights along the feed-forward structure are strongly decreased (Fig 3A), explaining why a propagation of activities is not taking place anymore (Fig 2D, right). This also becomes evident when assessing the weight change depending on the pre- and postsynaptic index (Fig 3D), in which the spatial distance between place cells corresponds to the distance to the main diagonal. We see that most of the weight changes are negative and occur in the feed-forward structure—that is below the main diagonal.

Overall, most synaptic weights in the stimulated, low-excitability network decrease over time (Fig 3G), implying that the memory representation is lost.

In contrast, in the unstimulated high-excitability network, we find that connections to close-by cells in the feed-forward structure remain at a higher level on average (Fig 3B). When examining the individual weight changes, we find that the connections at these distances split into two populations, one of which is potentiated beyond its initial level, whereas the other population decays. Accordingly, when examining the weight change depending on pre- and postsynaptic neuron index, we find large positive and negative changes close to the main diagonal, and mostly negative changes further away (Fig 3E). The origin of the two population becomes evident, when examining the initial weight of the connections: almost exclusively initially strong synapses being above a certain threshold (Fig 3H, dotted line) are potentiated, whereas those below the threshold decay. Thus, in the unstimulated high-excitability case, only the strongest synapses of a memory are potentiated by the rest phase and, thus, consolidated such that in the end a sparse backbone of the memory remains.

In the stimulated high-excitability network, on the other hand, a strong distinction in the temporal evolution of initially weak or strong weights is missing (Fig 3I). Accordingly, potentiated synapses can be found also for connections to more distant place fields (Fig 3C) and, hence, further away from the main diagonal (Fig 3F). Whereas initially, mostly strong synapses potentiate, also very weak synapses can undergo strong potentiation throughout the rest phase. Thus, the feed-forward structure recruits and strengthens existing weak synapses at larger distances, thereby making replay more reliable and speeding up the replay (compare Fig 2F, middle and right; Fig A:E in S1 Appendix), which is in strong contrast to the sparsification of the memory structure in the unstimulated network. The large distance synapses, however, make the replay less ordered leading to a decrease of replay quality (Fig A:F in S1 Appendix).

Intuitively, these phenomena can be explained by the fact that the synaptic weight determines the probability with which a postsynaptic neuron spikes after a presynaptic spike. Due to the synaptic delays and the integration window of the active dendrites, a postsynaptic spike that is being triggered this way, would have a temporal difference of around 6ms, which entails synaptic potentiation according to the STDP rule (Fig 1C). Thus, the probability of triggering such a postsynaptic spike regulates the balance between synaptic potentiation and depression, which ultimately determines whether a synapse will grow or decay over time. For the unstimulated, high-excitability network, most replays evolve from individual spikes within the feed-forward structure. Hence, the postsynaptic spiking probability is tweaked only by a single spiking presynaptic neuron and the weight of the respective connection and the initial weight of that connection essentially determines whether it is potentiated or not. On the other hand, further down the feed-forward structure or in the stimulated case, multiple presynaptic neurons may spike at the same time and collectively trigger a postsynaptic spike, such that the potentiation of each individual synapse does not depend solely on its initial weight.

### Analysis of activity propagation and plasticity

**Fig 4:**
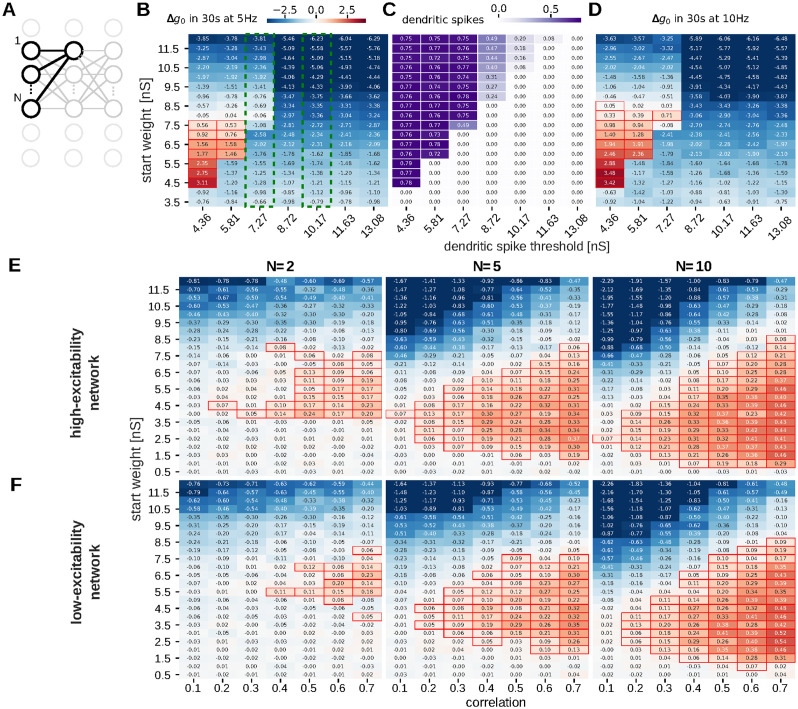
Results from a reduced model of plasticity dynamics in feed-forward structure. (A) Reduced model for studying parameter dependency of potentiation at a single postsynaptic neuron within a feed-forward structure with *N* neurons at every stage. (B-C) Change in synaptic weight as measured by conductance *g*_0_ (color-coded) of a single synapse with Poisson input with 5 Hz (B) or 10 Hz (D) for different initial weights (y-axis) and thresholds for non-linear dendritic integration (x-axis). Potentiation is additionally highlighted with red bounding boxes. Green dashed lines highlight the dendritic thresholds for low and high excitability. (C) Number of dendritic spikes within the simulations from panel B. Large weight changes occur only in the regimes where dendritic spikes are prevalent. The smallest weights for which potentiation occurs increase for increasing dendritic thresholds. Above a certain initial weight, depression is observed, which can be attributed to synaptic scaling. Hence, there is a stable fixed point at a high synaptic weight, corresponding to a consolidated connection. The transition between LTP and LTD occurs at smaller weights, when cells are more active. (E-F) Dependency of weight change on initial weight (y-axis), correlation between pre-synaptic Poisson spike trains (x-axis) and the number of such spike trains (columns, *N* indicated in title). For the conditions of the spontaneously active network (panel E), positive weight change occurs already for medium weights and for small numbers of presynaptic neurons and small correlation, whereas networks with low excitability need higher correlations or weights (panel F).

To provide a better understanding of the influences of different parameters on observed network dynamics, we study plasticity and the propagation of activity within the feed-forward structure in a reduced model. We consider a single postsynaptic neuron, which receives input from *N* presynaptic neurons that are located one “stage” further up the feed-forward structure (Fig 4A). This model allows us to control all initial synaptic weights to the post-synaptic neuron as well as the correlation in the firing of the presynaptic neurons.

We started with one presynaptic neuron and a single synapse. We quantified the influence of both its starting weight and the dendritic excitability, as evaluated by the current threshold for nonlinear dendritic activity on the final synaptic weight after 30 s. At low dendritic thresholds, small weights undergo weak depression (Fig 4B), because there is almost no post-synaptic spiking and, hence, no plasticity. Intermediate weights undergo potentiation whereas very high weights undergo depression (Fig 4B). In more detail, when the weight is strong enough to trigger postsynaptic firing, it will likely be potentiated by spike-timing dependent plasticity. As non-linear dendritic integration facilitates post-synaptic firing, the dendritic threshold has a strong influence on this. In particular, the minimal initial weight that exhibits potentiation increases with the dendritic threshold. When tracking the fraction of postsynaptic spikes that were preceded by a dendritic spike (Fig 4C), it becomes evident that potentiation only occurs when the initial weight is strong enough to trigger dendritic spikes. On the other hand, too strong weights will make the postsynaptic neuron fire too often. In this case the weight will decrease due to synaptic scaling. Thus, as predicted analytically in [43], the quadratic weight dependence of our scaling rule entails a stable upper fixed point for the synaptic weight. For spiking plasticity models, as used here, this stable fixed point becomes a stationary value around which the weight fluctuates: while pre-post-spike pairs slightly increases the weight, scaling decreases it in between. Fig 4B demonstrates, that also for our model there is such a stationary value between 7.5 nS and 8 nS to which all sufficiently strong synapses will converge (there is potentiation for initial weights below and depression above this value). We also observe such an upper fixed point in the network simulations with a slightly higher value (Fig 3H and 3I). Note, that this stationary value is independent of the dendritic excitability. However, the fixed point vanishes when it lies below the dendritic threshold (here between 7.27 nS and 8.72 nS). In that case, the synapse cannot trigger dendritic or postsynaptic spiking and only decreases over time. When repeating the simulation with an increased presynaptic rate, we obtain a slightly higher fixed point for the weight (between 8 nS and 8.5 nS; Fig 4D) in agreement with the above described analytical results [43]. The lower boundaries for potentiation are again determined by the dendritic excitation and not affected by the higher input rate.

We conclude that the potentiation of memories in feed-forward structures, where each postsynaptic neuron receives only inputs from one presynaptic one, strongly depends on the initial synaptic weight. Feed-forward structures, where each neuron only connects to one active presynaptic neuron will only potentiate high initial weights whereas connections with low weights decay, such that the memory representation becomes sparser. This is likely the case in the (unstimulated) high-excitability network.

However, as explained above, the feed-forward structures entail that there are often multiple presynaptic neurons that can be expected to fire at the same time or at least in a correlated manner. To investigate the influence of such correlated firing, we again selected two distinct dendritic sensitivities (Fig 4, green dashed regions), and vary the number of presynaptic neurons as well as the (instantaneous) correlation of their spike-trains along with the initial weight. We then evaluated the average weight change of the respective synapses. Here, with increasing number of presynaptic inputs, the correlation and the initial synaptic weights that trigger potentiation can be smaller (Fig 4E and 4F). This is explainable by the fact that dendritic spiking is determined by the in-flowing current in a short time interval and hence only depends on the product of weight and number of synchronously arriving spikes, whereas the expectation value of the latter is the product of the correlation and the number of inputs. As a consequence, feed-forward structures where each neuron connects to multiple synchronously active presynaptic neurons can potentiate (consolidate) also small synapses. As the external stimulation introduces such synchronous firing right from the start of a replay sequence, the stimulated high-excitability network potentiated a broad range of synapses and, thereby, strengthens its feed-forward structure.

### Transient increase in dendritic excitability reshapes and stabilizes memories

**Fig 5:**
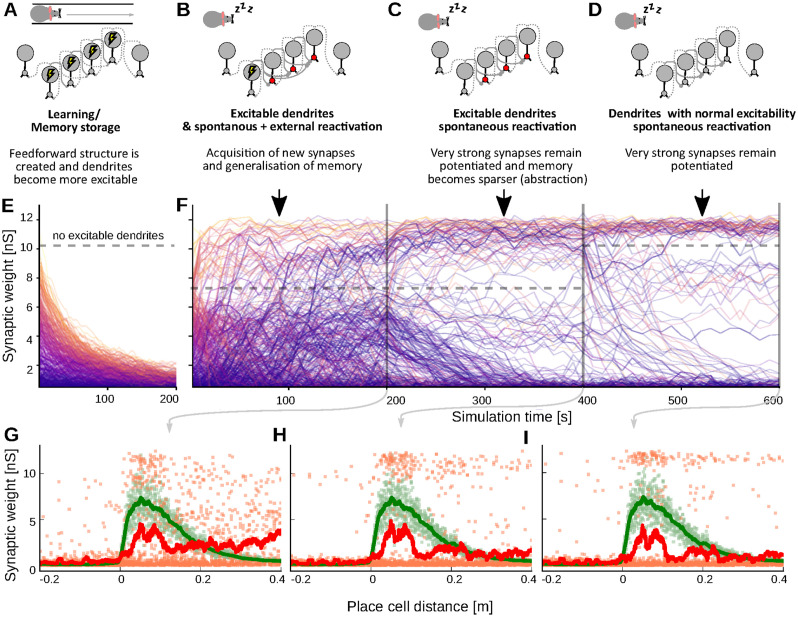
Hypothesized progression in the consolidation of episodic memories. (A-D) Schematic of the proposed progression: Immediately after learning (panel B), dendrites become more excitable (red circles) and external stimulation triggers replays (lightning symbol), such that also weak, memory-related synapses are potentiated. Later-on, the external stimulation ceases (panel C), but dendrites still have enhanced dendritic excitability, such that all sufficiently strong synapses continue to grow. Finally dendritic excitability decreases, but the feed-forward structure is sufficiently potentiated to still spontaneously reactivate and, thereby, maintain strong synaptic weights. (E) Time-evolution of synasptic weights without stimulation or enhanced dendritic excitability. All weights decay. (F) Time evolution of synaptic weights in a network undergoing the progression in panels A-D. After transient stimulation and enhanced dendritic excitability, the memory is strong enough to sustain itself. Solid grey lines indicate transition between phases whereas dashed grey lines mark the dendritic spiking threshold of the respective phase. (G-I) The weights after the different phases in B-D mapped to the distance between the pre- and postsynaptic neurons place-field. The feed-forward nature is preserved.

Taken together, the above results show that reliable activity propagation and related synaptic potentiation are possible for enhanced dendritic excitability, but also for a lower dendritic excitability in combination with strong synaptic weights. This fits well with the hypothesis that memories are primed for consolidation by a transient increase of dendritic excitability after learning [24]: Such a transient increase—maybe in combination with external stimulation—could potentiate and strengthen a freshly learned feed-forward-structure further such that it will afterwards have sufficiently strong weights to exhibit spontaneous replays and, thus, remain potentiated after the dendritic excitability returns to its normal level (Fig 5A–5D).

We tested this in a simulation with three phases: First, we run the network for 200 s with enhanced dendritic excitability and stimulation. As expected, we observe further strengthening and the potentiation of weak synapses within the feed-forward structure during this phase (Fig 5F and 5G). Second, we switch off the stimulation and run the network for another 200 s. Here, strong synapses are further strengthened, whereas intermediate and small weights decay (Fig 5F and 5H). Finally, we also decrease the dendritic excitability back to normal, low values. Whereas a few more synapses are decaying, the majority of synapses remains strong for another 200 s (Fig 5F and 5G). In contrast, when assessing the evolution of the initial feed-forward structure under normal dendritic excitability without the first two phases (Fig 5E), all weights decay. Hence, the transient increase in dendritic excitability (and the external stimulation) can indeed make a memory strong enough to undergo spontaneous reactivations and remain stable throughout the third phase. Thus, phases with enhanced dendritic excitability contribute to restructuring the memory and stabilize or rather consolidate it.

## Discussion

In summary, we have shown that fast replays of a sequential memory encoded by a feed-forward structure can lead to different patterns of synaptic plasticity, where the excitability of nonlinear dendrites plays a strong role. Dendrites with low excitability entail less reliable propagation of activity through the feed-forward structure, which, in turn, leads to a depression and decay of the weights of the memory—that is, no consolidation. When the dendritic excitability is high, memories can be consolidated, but in qualitatively different ways depending on external stimulation. On the one hand, without external stimulation, a backbone of the feed-forward structure with the strongest weights is further potentiated, such that a sparser representation of the memory emerges. This can be considered as a minimal and most energy-efficient representation that enables a reliable sequence replay. On the other hand, with external stimulation present, also small weights as well as connections to cells further “down” the sequence are potentiated, which can be interpreted as a form of generalization of the sequential memory. Note that this generalization goes along with a loss of temporal information as compared to the original memory. In the most extreme case, a spiking in a group of neurons triggers all neurons further down the sequence at once. Accordingly, there is a strong decline in quality of temporal structure of replays (Fig A:F in S1 Appendix). Hence, to preserve memory structure, consolidation by spontaneous replays is more optimal. In general, temporal structure is expected to be preserved when spikes of neurons with far separated receptive fields do not coincide within the potentiation window of the STDP-rule. In the stimulated case, this could be achieved by temporally very constrained stimulation that triggers only one volley of synchronous spikes that propagates through the structure (comparable to the test-pulses in Fig A in S1 Appendix, see [42] for an analysis).

In most computational models, replay relies on some form of stimulation and naturally stops as soon as the stimulation ceases. The spontaneous replays in our model would continue and need to be suspended when processing new experiences during wakefulness. This could happen through increased inhibition due to the ongoing processing, decreased neural (or dendritic) sensitivity [50], or a decreased strength of the excitatory connectivity (e.g., due to neuromodulation, [51]).

As an explanation for the different consolidation behavior in the different cases, we demonstrated that activity propagation and plasticity strongly depend on the dendritic excitability as well as on the number and the correlation of presynaptic neurons at the previous “stage” of the feed-forward structure (Fig 4). Although the correlations considered in our reduced model are very high, it is conceivable that such high correlations can be reached through common input of these cells. Also, gap junctions between groups of excitatory neurons could enhance correlated firing [33–35, 52]. Moreover, it can be expected that each “stage” of the feed-forward structures in the brain comprises a much larger amount of neurons, such that small synapses may be recruited even at much lower correlations.

In our model, sequence replay and memory consolidation only emerge when the dendritic non-linearity can be triggered by a few or even one (for the unstimulated model) strong synaptic inputs. In particular, our model synapses converge to 10 nS, which seems very large as compared to experimentally observed values, e.g. in CA1 [53]. In our model, the value of these stationary weights could be decreased by a weaker STDP-contribution or stronger synaptic scaling [43]. In that case activity propagation would still be possible if neurons were more sensible. One possibility we investigated here (Fig 4) would be lower thresholds for dendritic spiking. Moreover, the integration window for active dendrites could be increased up to 3 ms [54], which would increase the responsiveness to correlated, but temporally spread-out inputs. Additionally, also the membrane potential might (on average) be closer to the firing threshold, rendering the neuron more sensitive to synaptic inputs. Interestingly, a recent biophysical study [55] provided evidence that the latter is indeed the case *in vivo*, where dendrites receive a lot of background input. Thus, assuming that our model represents only part of the the hippocampal network with all other neurons subsumed into the background currents and the dendritic non-linearity, the derived requirements for consolidation may indeed be fulfilled in biological networks. Furthermore, also homeostatic processes that down-regulate neural firing rate may be suspended during sleep ([50], but see [56, 57]) for evidence on sleep homeostasis), which would make the neurons even more prone to firing. Moreover, in many cases neurons are connected by multiple contacts [58–63], which would be subsumed under a single, but much stronger synapse in our model (for an explicit implementation see [52]) and have a even higher chance to trigger non-linear dendritic integration. Thus, in summary a strong influence of a single synapse on dendritic and neuronal firing is indeed biologically plausible.

We also found that the propagation of activity through the feed-forward structure is facilitated by the synchronous firing of multiple neurons at each “stage” of a feed-forward structure. Similar results have been obtained in multitude of previous modeling studies [40, 64–67]. It can be expected that in a larger networks with larger feed-forward structrures, the number of synchronous neurons per stage increases, which in turn decreases the synaptic weighs required for activity propagation which were discussed above. Along this line, especially in random networks, active dendrites seem to foster the propagation of synchronous firing [68, 69]. Note that connections may in general be distributed over multiple dendritic arbors such that our model with its single dendritic compartment overestimates the amount of coincident inputs. Yet, on the long run, synapses can reorganize [70], and coactive synapses can be expected to cluster at the same dendrite (see, e.g. [71, 72]) exploiting local cooperative plasticity mechanisms (see, e.g., [73, 74]).

Our results now relate the mode of activity propagation to properties of the consolidated memory predicting that input-induced replays will lead to a generalized and strengthened memory representation that recruits and strengthens initially weak synapses during consolidation. Moreover, replays that are induced by an input at the beginning of the feed-forward structure, propagate through the whole structure and are, thus, more long-lasting than spontaneous replays which can start anywhere in the feed-forward structure. Strikingly, experiments show that longer replays are crucial for memory consolidation shortly after learning [49]. Thus, input-driven replays may be more prevalent shortly after learning, when additional, initially weak synapses need to be allocated to the memory. Eventually, however, the input stimulation for the respective feed-forward structure may decrease. In that case, our model predicts that the memories evolve towards a sparser, and thus more energy-saving representation. Such a sparsification may correspond to forgetting certain details and abstracting a memory to its gist, which is well known effect of sleep [75].

We therefore propose that the different synaptic plasticity dynamics that we found for different stimulations and dendritic excitability all contribute to the evolution and maintenance of a memory in different phases as both are transiently increased after learning. This is in line, both with the idea that dendritic excitability primes memories for consolidation [24] as well as findings that hippocampal memory is becoming independent of input only over time [76]. We tested this idea in an example setting with three phases with different dendritic excitability and external stimulation and showed that the memory was consecutively stabilized and generalized, then sparsified and pruned, and finally remained stable under basal conditions.

Thus, in summary, our model predicts that dendritic excitability and external stimulation jointly control replay activity during rest phases and their transient increase after learning can lead to functionally different phases of memory reorganization.

The model we used to obtain these results has been based on and extended from [42], which had been proposed to resemble CA1. The model components are hereby not as biologically detailed as possible, but instead the model is formulated such that it accounts for replay and ripple-like activity, but contains a reasonable mathematical complexity enabling simulations for variable cases. Along this line, the main components required for this kind of activity are (i) a mechanism that fosters activity propagation along a sequence of neurons—here the non-linear dendritic integration—and (ii) the excitatory couplings between the cells that can encode the sequence memory. Concerning the latter, experiments show that excitatory connections between pyramidal neurons in CA1 are far less abundant than the connection probabilities assumed in the model ([53, 77], but see [78]). Yet, the network model used here and in [42] only represents a sub-sample of the actual network. Therefore, to observe large connected sub-networks (e.g., feed-forward structure), that are present in large networks with smaller connection probability by chance, higher connection probabilities must be used in the model. On the other hand, the model is abstract enough to be applied to other networks such as CA3, which has abundant recurrent connections [79] and is also known to be able to generate ripple activity [7, 80–82] and sequential replays [81]. Moreover, also CA3 exhibits fast dendritic sodium [83] as well as NMDA spikes [84]. Therefore, it is conceivable, that the mechanisms discussed here can be observed in CA3 with external inputs arising from dentate gyrus [85] or entorhinal cortex [7].

## Supporting information

S1 File Simulation code. Jupyter notebooks with the routines used for the described simulations.
(ZIP)

S1 Appendix Assessment of replay quality. (PDF)
