# Supplementary material for: Differences in the consolidation by spontaneous and evoked ripples in the presence of active dendrites
Source: PLoS Comput Biol. 2024 Jun 25;20(6):e1012218. doi: 10.1371/journal.pcbi.1012218 (PMC11230591; doi:10.1371/journal.pcbi.1012218)
Supplement: S1 Appendix — (PDF) [file pcbi.1012218.s002.pdf]

Supporting Information - Replay quality

To evaluate the quality of sequence replay, we use the spike-trains of all neuron pairs i and j in the feed-forward structure and evaluate how often neuron i spiked in a 50 ms-window before neuron j. If $i < j$, this number is added to the number of forward pairings n_{forward}, whereas when $i > j$ it is added to the number of backward pairings n_{back}. We then use the matching index [1]

$$I = \frac{n_{\text{forward}} - n_{\text{back}}}{n_{\text{forward}} + n_{\text{back}}}$$

to quantify replay quality.

First, we evaluated this index in five 400 ms intervals every 10 s (Fig A:B/D/F, green curves). As in our model, stimulation is implemented by noise-modulation, the neurons in the stimulated group fire asynchronously during the peaks of the stimulation (Fig A:A,E, panels i/iii, yellow area; comparable to stochastic resonance) or even spontaneously (Fig A:C, panels i/iii yellow area). This leads to rather small correlations in firing. As a consequence, the activity propagation along the feed-forward structure is also rather asynchronous implying that the stimulated neurons that fire first already trigger neurons further down the sequence before slower stimulated neurons fire (Fig A:A/C/E, panels i and iii). This, in turn, increases n_{back} and yields small matching indices (Fig A:B/D/E, green curves). Moreover, in cases with spontaneous ripples (high excitability), new replay events can start while activity still propagates at the back of the feed-forward structure. This leads to even more backward spike-pairs and thus lower matching indices (Fig A:D). It can also be seen that, if replays are induced by stimulation, their matching index is initially higher but decreases over time as plasticity reorganizes the memory representation (Fig A:B/F). This is because the replay becomes less sequential, but rather all neurons in the feed-forward structure are triggered at once through spikes of the stimulated neurons.

Second, for better understanding of the activity propagation and comparability with previous models [1], we ran another set of simulations: We introduced time-intervals, where plasticity is turned off and the stimulated group, even in the spontaneous case, receives distinct current pulses (Gaussian with std. 1 ms, -0.15 mV offset and 6 mV amplitude at a frequency of 5 Hz for 1 s every 10 s). We then evaluated the matching index in 50 ms intervals after these pulses. For such pulse-induced replay events, the stimulated neurons fire more correlated (Fig A:A/C/E panels ii and iv), although the firing correlation is still not instantaneous. However, this synchronized volley of activity leads to more synchronous spike groups as it propagates down the initial feed-forward structure in a very ordered fashion (panels ii), which leads to much higher matching indices (Fig A:B/D/F, blue curves). For the high-excitability case without stimulation, this behavior is preserved and the replay quality remains high (Fig A:C-D). For the stimulated case with high excitability, plasticity increases the weights to neurons further down the feed-forward structure, such that a test-pulse triggers one or two synchronous spikes of the whole structure at the end of the simulation (Fig A:E, panel iv). As the are not 100% synchronous and some neurons randomly spike before others, this also increases the number of backward spike-pairings and lowers the matching index over time (Fig A:F). For the stimulated case with low excitability, only a few synapses retain high weights and the pulse-induced spikes only induce a few spikes along the feed-forward structure, which leads to a low replay quality (Fig A:F).

References

1. Jahnke S, Timme M, Memmesheimer RM. A Unified Dynamic Model for Learning, Replay, and Sharp-Wave/Ripples. Journal of Neuroscience. 2015;35(49):16236–16258. doi:10.1523/JNEUROSCI.3977-14.2015.

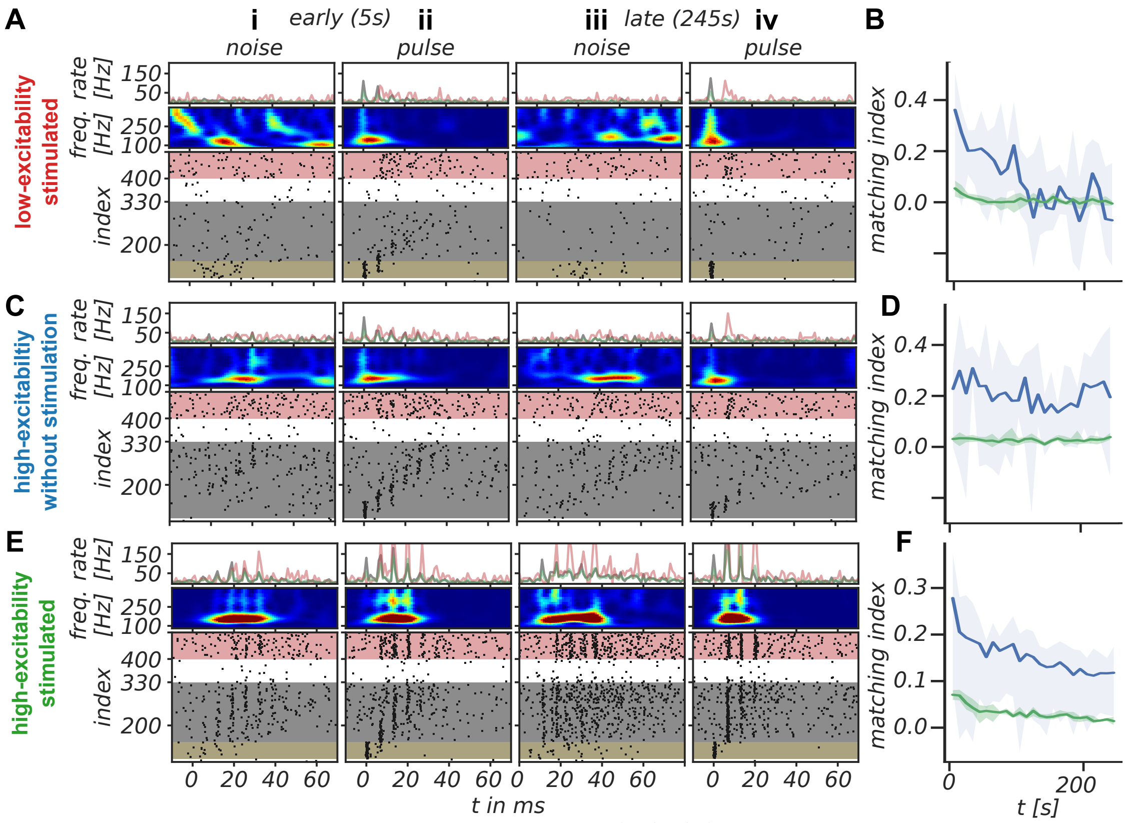

Fig A. Replay in the different model variants (A/C/E) Replay events during early (around 5 s, panels i-ii) and late (around 245 s, panels iii-iv) simulation. Panels (i) and (iii) depict replays during normal simulation (noise-induced), while panels (ii) and (iv) show relays induced by test pulses. (Top) Mean rates of feedforward (gray) and inhibitory (red) population and all neurons (green). (Middle) Time-dependent CWT (colors scaled to max. coefficient in window) (Bottom) Spike-rasterplot. (B/D/F) Time-evolution of matching index calculated from 5 test pulses (blue) and five 500 ms windows (green). Solid depict the mean while shaded areas mark minimal and maximal values.
